# Glanzmann’s thrombasthenia and molecular mimicry

**DOI:** 10.4103/0971-6866.64942

**Published:** 2010

**Authors:** Viroj Wiwanitkit

**Affiliations:** Wiwanitkit House, Bangkhae, Bangkok Thailand 10160. E-mail: wviroj@yahoo.com

Sir,

I recently read with great interest the current publication of Ghosh *et al*.[1] It is interesting that the antiplatelet antibodies can also be present in patients who had not received any platelet transfusion. In this regard, the molecular mimicry might be the responsible mechanism as the author’s proposal. Definitely, there must be a previous stimutation, which is assumed to be an autoantigen that can lead to the generation of antiplatelet antibodies.[2] The other proposal regarding the possibility of another mechanism seems to be a weaker hypothesis. Considering that the author had performed well control on the laboratory analysis system, there should not be other interference on the analysis.
